# The Impact of Dynamic Seating on Classroom Behavior of Students with Autism Spectrum Disorder

**Published:** 2017

**Authors:** Nader MATIN SADR, Hojjat Allah HAGHGOO, Sayyed Ali SAMADI, Mehdi RASSAFIANI, Enayatollah BAKHSHI, Hossein HASSANABADI

**Affiliations:** 1 Depatment of Occupational Therapy, University of Social Welfare and Rehabilitation Sciences, Tehran, Iran; 2 Department of Life and Helath Science, University of Ulster, UK; 3 Pediatric Neurorehabilitation Research Center, University of Social Welfare and Rehabilitation Sciences, Tehran, Iran; 4 Department of biostatistics, University of Social Welfare and Rehabilitation Sciences, Tehran, Iran; 5 Department Physical Medicine and Rehabilitation, University of Medical Science, Mashhad, Iran

**Keywords:** Autism spectrum disorder, Students, Dynamic seating, Iran

## Abstract

**Objective:**

Children with autism have sitting and on-task behavior problems in class. In this study, the effect of three alternative classroom-seating devices such as regular classroom chairs, therapy balls, and air cushions were examined on students’ classroom behavior.

**Materials & Methods:**

15 students with autism participated in this A1-B-A2-C multiple treatments study from Mashhad’s Tabasom School, Mashhad, Iran in 2014. Students’ behaviors were video recorded in three phases: sitting on their common chairs during phase A, air-sit cushioned in phase B, and ball chairs in phase C. Sitting times and on-task behaviors were quantified by momentary time sampling and compared during different phases for important changes during 8 wk. Additionally, the Gilliam Autism Rating Scale-Second Edition test was used to examine stereotyped movements, social and communication skills of the students in the before and after research.

**Results:**

Significant increases in in-seat behaviors in 86.7% (thirteen out of 15) of the students and on-task behaviors in 53.3% of the students (eight out of 15) when seated on therapy balls. Air cushions had no significant effects on in-seat/on-task behaviors. The results also showed significant decrease in stereotyped movement and increase in communication and social skills of these students. The teachers also preferred the use of the balls and/or air-cushioned chairs for their students.

**Conclusion:**

Therapy ball chairs facilitated in-seat behavior and decreased autism related behavior of the students with Autism Spectrum Disorder in class.

## Introduction

The overall population of Autistic Spectrum Disorder (ASDs) is 14.7 per 1000 (one in 68) in children aged 8 yr old (1). In Shiraz/Iran 1.9% of children had been diagnosed with ASD (2). Based on the evidence reviewed, the median of prevalence estimates of autism spectrum disorders was 62/10 000 (25). A report in 2014 in Iran represents a prevalence rate of 95.2 per 10,000 (25). Similar findings reported in 2015 in Iran (26). Hence, the increasing rate of ASD prevalence is a great challenge for the education system and necessitates national efforts to eliminate consequences. 

Children with ASD exhibit inattention and distractibility more than normal children (3, 4). These children confront various academic problems, such as difficulties in class participation, low attention span, and inappropriate behaviors which hinder their ability to take part in educational activities (5). They are distracted easily from education by their own repetitive, restless, and disruptive classroom behaviors. These students usually experience disappointment in educational progress with usual intervention strategies, since these strategies do not deal with the sensory issues that may diminish the upsetting behavior (6, 7). 

One main characteristic of children with ASD is their problems in sensory processing and sensory integration which negatively impact their engagement in daily activities (8). Sensory integration deficiencies in these children results in poor engagement in academic tasks (9). 

Each child has a different model of sensory processing insufficiency involving tactile, proprioceptive, vestibular, visual, and auditory processing which may lead to sensory seeking and finally affect his/her educational achievement (10). Avoiding tactile experiences and reduced motor responses due to hypersensitivity or hyposensitivity to touch input needs more powerful, or numerous tangible inputs for regulating their arousal level (9). Everybody needs efficient sensory inputs to organize an adaptive behavior, which would be helpful in providing the best conditions for learning (5). When specialists use appropriate programs to supply children who need sensory stimulation, these children participate actively in daily living performances because they maintain arousal states for adapting behavior and reacting to environmental challenges (10). Nowadays, occupational therapists have focused on adjusting the environment for ASD students to help them achieve academic improvements (3). 

The environmental modification in the classroom can increase students’ engagement in academic tasks (11). Accordingly, the relationship between the student and the classroom environment needs to be better clarified in order to facilitate educational performance for all students in inclusive classrooms (3). Ecological systems viewpoints express the risks or benefit that affect the relations between the student and the environment (12). 

Therefore, ignoring proper changes in environment may have severe negative impacts on the learning processes (3). In normal situations, students usually sit on school chairs for 5 hours in each single school day. This fact highlights the importance of using suitable remedy chairs and tables in schools. Children with autism need comfortable school chairs to gain success in educational achievements (3). Finding appropriate chairs is possible through investigating the impacts of different changes in seats on academic performances of ASD students, because providing different chair choices in class improves their academic experiences (13). 

The application of the therapy balls as an alternative chair may provide chances for the students with sensory integration deficiency to settle better on chairs in class and engage in the class task (9). On the other hand, sitting on balls as chairs has its own limitations such as occupying a large space in class, which may hinder its application in small classes as well as the need for carpet under the ball. In addition, to ball sitting, air cushions can also be used because they have some advantages, such as being cheaper (14) or more stable compared to therapy balls. Therefore, if air cushions have competitive therapeutic effects, their usage in classrooms would be better than ball sitting. 

Some questions were address in this study as follows: Which type of seating (chair, air cushion, or ball) is preferred to be used by the teachers? To what extent will dynamic seating chairs affect on-task/in-seat behaviors in a special education classroom? To address these questions, the impacts of sitting on a chair, ball, and/or air cushion on in-seat/on-task behaviors were investigated. 

## Materials & Methods

**Research Design**

A single-case, multiple treatment A1-B-A2-C design was used to explore the influence of three seating options, including typical chairs, therapy balls, and air cushions on in-seat and on-task behaviors of 15 students with ASD. During the two A phases, all students sat on typical chairs. Then, they sat on either cushions or balls, each for 2 weeks. To tackle the effect of intervention order, in phase B, eight students were seated on air cushions and seven on balls, and during phase C, the ball and air cushion groups were changed with each other (cross-over); that is, the students who sat on balls in phase B sat on air cushions in phase C and vice versa. To remove any novelty effects, the students were initiated to use stability balls and air cushions instead of their chairs for 2 days, before baseline data gathering.

**Participants**

In four classes, 15 participants who studied naturally were selected with convenience sample from the preschool students in an autism elementary school (Tabasom) in Mashhad, Iran in 2014. All the students took their own routine medication during the research period. According to their teachers’ reports, most of children were identified as having difficulty with inseat and on-task behavior. The inclusion criteria were ASD students aged between 7 and 10 yr. The exclusion criterion was any physical disability interfering with sitting on a ball or a cushion.

**Procedures**

All the participants’ parents were provided with the information sheet and ensured that their participation in the research was voluntary, and that they were able to withdraw from the study at any stage of the data collection process. Following their consent, data were collected in the participants’ convenient time and day with ethics approval code: USWR.REC.1392.118 on 5th November 2013. All students with ASD provided consent included in the study.

**Data Collection**

**Video Recording**

Camera recorders were set in the class and the students’ behaviors were recorded during class tasks. Assessments were done three times per week every other day, with an overall of 24 sessions for all classes. Each participant was monitored for 10 min per session. Their behaviors during class time (sitting times and on-task behavior) were recorded by a video camera. Two intervention phases were compared with the child’s baseline and withdrawal phases. Two teachers were trained as spectators of video films. In-seat and on-task behavior data were gathered via momentary time sampling (MTS). The spectators coded the student’s behavior at 10 sec intervals individually, stopped the video, and marked the interpretations on each child in the allocated table in each session; therefore, 60 observations per session per participant resulted. These MTS intervals were provided to make the observations more valid and representative of the child’s behavior throughout the baseline and interventions periods. Two coding categorizations permitted the observers to obtain in-seat and on-task behaviors of students. The teacher gave no extra feedback on students’ sitting and on-task behaviors during the research. However, if a student had a behavior that could possibly be dangerous to him/ her, peers, or the teachers, it had to be prevented by the teachers. 

Video records were regularly checked throughout the study by two observers to determine inter rater reliability agreement for at least one session per phase for each of the participants. Inter rater agreement percentages ranged from 95% to 100% for in-seat behavior and from 85% to 100% for on-task behavior. This inter-reliability ranged from 88% to 100% (15).

**On-Task Behavior**

Participation was described as “oriented towards appropriate classroom activity or teacher and either interacting with materials, responding to the speaker or looking at the speaker.” (5). This definition included writing as well.

**In-Seat Behavior**

Data of in-seat behavior were defined as: any of the child’s buttocks to get-in-touch with the seat segment of the chair and all legs of the chair to get-in-touch with the floor (5). For the intervention phase (B), any part of the student’s buttocks in contact with the air cushion, the air cushion to get in touch with the seat segment of the chair, all the legs of the chair to get-in-touch with the floor (16). For the intervention phase (C), in-seat behavior was defined as any segment of the student’s buttocks to getting in touch with the ball, the ball to getting in touch with the floor, and at least one foot to getting in touch with the floor (5). 

The researchers considered on-task and in-seat selections, because the students might have been on-task but out of the seat, cushion, or ball. Conversely, the students in sitting positions might have been doing stereotyped movements.

**Teacher Social Validity Scale**

A social validity questionnaire was used at the last part of the study to evaluate the teachers’ satisfaction about the interventions. The questionnaire involved 10 questions and assessed the impacts of the intervention on sitting and engagements, in addition to preference among therapy balls, air cushions, or chairs. Questions were answered on a 5-point Likert scale that ranged from strongly disagree [1] to strongly agree [5].

**GARS II (Gilliam Autism Rating Scale-Second Edition)**

The GARS II test was fulfilled by a trained administrator to examine the social skills, the stereotyped movements, and the communication of the students in baseline and final phases for all participants. The GARS II is a screening instrument used for the assessment of individuals aged 3–22 yr who exhibit behavioral characteristics that may be indicative of autism. This is a standardized instrument, which consists of 42 items divided into three subscales that describe specific observable and measurable behaviors (17). The items included in this instrument are based on the definition of autism adopted by the Autism Society of America and on the diagnostic criteria for autistic disorder published in DSM-IV-TR. GARS II, because it is validated and standardized on Iranian population. In Iran, this scale’s utility is based on Samadi, and McConkey (17).

**Materials for Intervention**

Therapy balls: The selected therapy balls used in the classroom had a diameter of 45 cm (Gymnic ball, Italy). Therapy balls were individually fitted for every student with specific amounts of inflation that confirmed the student could sit comfortably with his feet flat on the floor, with knees and hips flexed at 90 degrees. Each ball was labeled with the participant’s code. A bicycle tire was put under the therapy ball to provide stability and to prevent the ball from rolling too much. The inner diameter, outer diameter, and the circumference of the bicycle tire were 29.5, 41, and 129 cm respectively. It limited the sway distance of the ball to less than 2 cm. Air cushion: The Disc ‘O’ Sit cushion is round and filled with air. It is strong enough to sit on and is designed to fit on a classroom chair and provide movement while seated (16). 

Chair: A common wooden, iron frame classroom chair without armrests (height, 72; depth, 34; width, 39; seat height, 36 cm). 

Then the paired t-test with SPSS ver. 19 (Chicago, IL, USA) was used to analyze data. Normality of the data was approved by using Kolmogorov-Smirnov. Because of the repeated measurements method, the parametric test of Generalized Linear Model was used to decide on the significant differences between the interventions and baselines. 

## Results

**Demographic Data**

The sample included 15 participants. The ages of the participants ranged from 79 to 117 months with a mean age of 104.27 months ± 11.98. The participants were divided into four classes. Characteristic data included: (10 Males 66.7%, Females 5 33.3%), age 104.27±11.98 (months), Height 129.40±10.25 (cm), Weight 29.2±10.03 (kg). 

**Table 1:** The Effects of Different Seating on In-Seat Behavior

**Mean** *															
**Type of chair**	S1	S2	S3	S4	S5	S6	S7	S8	S9	S10	S11	S12	S13	S14	S15
**Chair1**	44.1	34.7	54.5	54.2	26	19	41.5	44.7	47.5	44.7	51.5	41.2	45.5	14.6	45.8
**Cushion**	35.4	22.2	58.5	53.2	19.2	46.3	40.3	51.2	52.5	51.2	43.8	38	58.6	45.6	57
**Chair2**	40.5	44.5	57.2	54	22.8	41.6	17.6	46.4	56	46.4	51.2	46.8	56	36.8	59.2
**Ball**	58.8	51	57.6	59.7	40	59.2	50.7	58.1	58.3	58.1	48.5	44.1	45	40.6	56.6

S= Student

* := (mean of 60 times observation per session, for six sessions during 2 wk

Positive changes in in-seat behavior were significant between A1 and C phases. Most participants (13 out of 15) demonstrated improvements in in-seat behavior when seated on a ball and these improvements were from 2.9 times in S12 to 40.2 in S6, with an average of 14.6. A2 and C Phases had an obvious difference in 11 students from 0.4 to 33.1 in S3 and S7 respectively, with an average of 11.6. The students’ in-seat behavior improved for S3, S6, S8, S9, S10, S13, S14, and S15 from 4 in S3 to 31 in S14, during the B phase (cushion). In comparison with A1 phase, most students’ in-seat behavior improved during the B phase. The students’ on-task behavior improved only for S6, S8, and S14 students, with 10.5, 23, and 0.1 times during the B phase (cushion) respectively in comparison with A1 phase. All other 12 students’ on-task behavior decreased from 1 to 22.8 times during the B phase. 

**Table 2:** Effects of Dynamic Seating on On-Task Behavior

**Type of chair**	**S1**	**S2**	**S3**	**S4**	**S5**	**S6**	**S7**	**S8**	**S9**	**S10**	**S11**	**S12**	**S13**	**S14**	**S15**
**Chair1**	21.4	24	34.8	31	24.7	16	40.1	18.5	30.7	7.7	10	38.2	41.5	45.4	29.6
**Cushion**	7.6	15	18.7	18.8	22.7	26.5	37.6	41.3	26	6.6	7.3	21.7	29.4	45.5	18.6
**Chair2**	14.5	29	44	24.8	22.2	11.2	26.2	34	28.6	7.6	12.2	28.6	42	39.8	26.8
**Ball**	20.6	33	25.6	28.2	22.7	28.7	50	34	35	1.3	5.5	17.6	25.3	32.3	40

S= Student

* := (mean of 60 times observation per session, for six sessions during 2 wk

The S1/ S2/ S4/ S5/ S6/ S7/ S9/S15 students’ on-task behaviors improved from 5 in S5 to 23.8 times in S7 during the therapy ball phase (C) in comparison with A2 phase. Therefore, employing air cushion led to an increased on-task behavior in few students and the ball in more than half of them in this study. 

**Table 3:** Distribution of on-Task/in-Seat Behavior (per Second) and GARS II of Participants in Different Phases (n=15)

**Type of chair**	**Assessment fields (Mean ±SD)**		
	**In-Seat behavior**	**On-Task behavior**	**GARS II**
**Chair1 A1**	40.1 ±19.6	27.8±16.2	43.4 ± 11.66
**Cushion B**	47.3±15	21.2±16.1	…..
**Chair2 A2**	43.6±17.7	25.4±16.5	…..
**Ball (C)**	51.2 ±13.6	26.1±17.5	39.5 ± 10.07

**Table 4:** P-value of in-Seat/on-Task Behavior of Participants in Different Phases (n=15

**In-seat behavior**		**On-task Behavior**	
A1-B	*P*= 0.82	A1-B	*P*< 0.058
A1- A2	*P*= 0.19	A1- A2	*P*= 0.77
A1- C	*P*< 0.001	A1- C	*P*= 0.44

As Tables 3 and 4 illustrate, the mean of in-seat behavior scores increased significantly from phase A1 with a Mean of 40±19.6 to a Mean of 51.2±13.6 in phase C. These results show noticeable improvements in inseat behavior during the utilization of therapy balls for classroom seating (P< 0.001). The change in the mean scores increased significantly from a Mean of 43.6±17.7 to a Mean of 51.2±13.6 (Table 4). 

Although, the mean of sitting increased from 40.1±19.6 in phase A1 to 47.3±15 in B phase, the air cushion did statistically not differ significantly from A1 phase in inseat behavior when comparing A1 with B phase. Despite improvements, on-task behaviors in most of the students (eight students) when sitting on balls in comparison with A2, dynamic seating had no significant positive effect on on-task behavior. 

The teachers preferred the use of balls and air cushions for students in class, according to the social validity questionnaire. The level of significance of the GARS II test was positive with (P= 0.017) and a Mean of 43.4 ± 11.7 in the beginning of the study, diminishing to a Mean of 39.5 ± 10.07 at the end of intervention.

## Discussion

The aim of this study was to compare the effectiveness of dynamic seating devices on improving a students’ in-seat and on-task behaviors and decreasing autism related behaviors within the classroom setting. Are there any differences between sittings on dynamic or ordinary chairs? 

In-seat behavior increased significantly when the students sat on therapeutic balls (phase C) when compared with sitting on normal chairs. This study proposes that ball seating in the classroom causes an increase of 86.7% in in-seat behaviors of ASD students with a P-value of less than 0.001. Therapy balls provide sitting and moving concurrently, which may satisfy sensory needs (18). In accordance with findings by Schilling et al, significant changes in inseat behaviors in Attention Deficiency Hyperactivity Disorder and ASD children have been reported when sitting on therapy balls (5, 19). Children with autism are supposed to select self-stimulatory behaviors, such as stereotyped movements to normalize arousal levels (14). As a result of normalizing arousal levels and regulating sensory inputs with rocking and bouncing on a ball, students with autism could be satisfied physiologically and would not need to engage in selfstimulatory behaviors (20). An agitated child may be relaxed by gently rocking on a ball (19). Therefore, stereotyped behaviors of the students with ASD might be decreased with Ayres’s sensory integration programs (21). 

Sitting on a cushioned chair had no significant effect on in-seat behavior, although it improved sitting times for most of the students. Sitting on the ball produced dynamic sitting that stimulated proprioceptive and vestibular systems (18). Vestibular and proprioceptive stimulation in ASD students who use dynamic seating can adjust arousal situations (3, 6). Unlike the ball, a cushion provides a more stable surface for sitting, and the children do not need more muscle activity to keep balance on seats. Therefore, they are less conscious about their balance, which may diminish their arousal level to sit calm and stay relaxed on a cushioned chair. Ball sitting was effective for most students in this research in the field of sitting times, but the results demonstrated unique responses of the students with ASD to the use of balls and cushions for sitting in the field of on-task behavior. Most ASD students (eight out of 15), showed a positive increase in on-task behavior while being seated on a ball, and this increase occurred only for a few students while using a cushion. Each type of furniture provides different effects depending on personal sensory needs of the students. Therefore, maintaining an optimal arousal state of each student depends on the type of furniture (3). Cushions provide a more stable seat; therefore, they cannot provide enough sensory stimulation to create functional benefits such as increasing on-task behaviors. 

Furthermore, the teachers’ reports supported the use of balls and air cushions for the students in class. Accordingly, the students were calmer when compared to the use of common chairs. Since agitation and attention deficiency seem to be due to sensory integration impairment, sitting modification in class condition may be beneficiary to students with autism (19). Children with attention problems are usually in low arousal condition; therefore, occupational therapists use a suitable intervention such as the “Disc ‘O’ Sit” cushion for the most favorable attention and education (16). 

Therapy balls and cushions can decrease behaviors related to autism of the students with an ASD significantly, according to the GARS II tests. The students feel better and more comfortable while sitting on therapy balls (22). Sensory processing may result in a decrease in social isolation and inattention to class tasks (23). Ball sitting also allows children to release their energy and receive sensory stimulation simultaneously. Thus, they do not need disrupting sensory seeking behaviors (24). 

When attempting to establish inclusive education classes for ASD children, changes to the schools’ environment to provide ideal interventions for these students are needed. The findings of this study show that different kinds of furniture have different results on ASD students. Understanding the priorities of the students and the availability of different chair choices might help in achieving more positive results, especially in the field of on-task behavior. Ball seating devices were found appropriate for most of the students to increase sitting times, but the results demonstrated unique responses of the ASD students to the use of cushions for sitting. Use of balls for sitting appeared to be positive in helping teachers in class to control destructive behaviors. The teachers reported that therapy balls and air cushions prevented disruption of class conditions, and their use also made students more socialized. They suggested that other teachers should apply these interventions for ASD students as well. The GARS II scores also approved a reduction of behaviors related to autism in students with an ASD in this study. As a limitation of this study, the duration of the treatment in this study was short (9 wk), and monitoring only four classrooms for the purpose of this research could be considered as another limitation. A bigger sample size and longer duration of time could possibly strengthen the results. Working as an interdisciplinary team is another necessary concern in inclusive schools.

**In conclusion,** the comparative researches help to recognize different aspects of features of alternative seating devices that are more suitable for specific sensory needs of the ASD children. With regards to thousands of students with special difficulty in sitting and classroom performance, these devices may be an optional selection for solving class behavior problems. 
